# Helicobacter Pylori DNA in Liver Tissues From Chronic Hepatitis C Egyptian Patients

**DOI:** 10.4021/gr356w

**Published:** 2011-11-20

**Authors:** Moushira A. Mahmoud, Loaa A. Tag Elden, Mohamed M. Awad, Henock A. Haile

**Affiliations:** a Department of Medical Biochemistry, Suez Canal Faculty of medicine, Ismailia, Egypt; b Department of Internal Medicine, Suez Canal Faculty of medicine, Ismailia, Egypt

**Keywords:** HCV, Chronic hepatitis C, *H. pylori*, Liver fibrosis

## Abstract

**Background:**

Hepatitis C virus (HCV) is considered the most common etiology of chronic liver disease in Egypt, which may progress to cirrhosis and hepatocellular carcinoma (HCC). Previous studies have documented an association between *Helicobacter pylori* (*H. pylori*) infection and liver cirrhosis with or without HCC. This study aimed to investigate the presence of *H. pylori* DNA in the liver tissue of Egyptian patients with chronic hepatitis C (CHC).

**Methods:**

Fifty-two CHC Egyptian patients were enrolled in this study. Plasma anti-*H. pylori* IgG was assessed with ELISA. Liver biopsies were tested for presence of *Helicobacter* DNA using genus specific nested polymerase chain reaction (PCR) and species was identified by sequencing.

**Results:**

Anti-*H. pylori* IgG was detected in 31/52 (59.6%) CHC patients while *Helicobacter* DNA was detected in 6 (11.5%) patients, all were *H. Pylori* by sequencing. *Helicobacter* DNA was more frequent in patients with high stage liver fibrosis (33.3%) than in those with low stage fibrosis (2.7%) (P = 0.006). There was no association between the presence of *H. pylori* DNA in the liver and age, gender of patients, liver function tests, AFP levels or viral load.

**Conclusions:**

These data confirm the presence of *H. pylori* DNA in liver of some CHC Egyptian patients and suggest an association of this bacterium with progression of liver fibrosis.

## Introduction

Hepatitis C Virus (HCV) infects an estimated 170 million persons worldwide. The prevalence of HCV infection varies throughout the world, with the highest prevalence (14.7%) reported in Egypt [1]. More than 70% of HCV-infected individuals develop chronic disease, which can progress to liver cirrhosis and hepatocellular carcinoma (HCC). The course of HCV related hepatic disease varies markedly from one patient to another. It is affected by age at exposure, duration of infection, alcohol intake, male gender, viral immune response [2]. However, even in the absence of these factors, disease progression may be observed in some patients, suggesting the role of other factors which remain to be identified. Host genetic factors or environmental factors, such as a bacterial co-infection, could be involved [3].

*H. pylori* is a Gram-negative organism that colonizes gastric mucosa and known to cause chronic gastritis, peptic ulcers, and gastric adenocarcinoma [4]. However, several separate research groups have detected *Helicobacter* spp. in liver tissue of patients with different hepato-biliary diseases. Nilsson et al have identified *H. pylori* in human liver samples from patients suffering from primary sclerosing cholangitis and primary biliary cirrhosis [5]. Tolia et al have demonstrated, by PCR, the presence of genomic sequences of *Helicobacter* spp. in the liver tissue of 40 patients with miscellaneous liver diseases; a further analysis by sequencing revealed that most of these species were *H. pylori* [6]. Several researchers reported that *H. Pylori* DNA can be found in patients with primary liver carcinoma and probably linked to the carcinogenic process in the liver [7, 8]. Moreover, studies from different countries documented the detection of *H. pylori* DNA in the liver tissue of patients with HCV-related chronic hepatitis, cirrhosis and HCC, suggesting that these bacteria could be implicated in the progression of CHC to cirrhosis and HCC [9-11].

In Egypt, a recent study demonstrated that the seroprevalence of *H. pylori* increased significantly in the HCV-infected patients when compared to healthy controls. Moreover, the researchers found that the prevalence of *H. pylori* infection increased significantly from chronic active hepatitis to cirrhosis [12]. However, up to our knowledge, the presence of *H. pylori* in the liver tissue of HCV Egyptian patients was not previously investigated. Therefore, this study was conducted to investigate the presence of *H. pylori* DNA in liver biopsies from Egyptian patients with CHC.

## Patients and Methods

Fifty two consecutive patients with CHC were included in the study. Those patients were required to have a percutaneous liver biopsy at the Gastroenterology and Hepatology Unit of Suez Canal University Hospital, as a line of their management. All were positive for anti-HCV and HCV RNA. Chronic hepatitis C was diagnosed by having either elevated or fluctuating ALT levels for more than 6 months and/or bright liver appearance on abdominal ultrasonography [13]. Patients with other causes of liver disease, including hepatitis B, were excluded from the study. The study was approved by the Research Ethics Committee of the Suez Canal Faculty of Medicine and informed consents were obtained from all study subjects. Liver function tests, AFP and anti-schistosomal antibodies were measured using commercially available kits. The HCV viral load was quantified using Real Time PCR technique in an ABI PRISM^®^ 7000 thermocycler (Applied Biosystems, Foster City, CA).

### Liver tissues processing

Each liver biopsy sample was divided into two parts: One was fixed in formalin and embedded in paraffin wax for conventional histological evaluation; the other was immediately frozen and stored at -80 °C until further molecular analysis. The formalin-fixed specimens were examined by pathologists at pathology department of Suez Canal University Hospital. Liver fibrosis was staged on a 0 - 6 scale according to Ishak score [14]. Patients were divided into two groups based on fibrosis score: “low stage” [F0-F3] and “high stage” [F4-F6] liver fibrosis.

### Detection of anti-*H. pylori* IgG

Plasma samples were tested for anti-*H. pylori* IgG antibody using a commercial test kit, AccuBind™ ELISA Microwells (Monobind Inc, Lake Forest, USA), according to the manufacturer’s instruction. Results were considered positive when higher than 20 U/mL.

### PCR amplification with Helicobacter genus-specific primers

DNA was extracted from frozen liver tissues using Wizard^®^SV Genomic DNA Purification System (Promega Corporation, Madison, USA). DNA quantitation was performed using the NanoDrop^®^ (ND)-1000 Spectrophotometer (NanoDrop Technologies Inc., Washington,USA). The extracted DNA was stored at -20 °C until further used. Nested PCR was performed with genus-specific primers for the Helicobacter 16S ribosomal RNA gene (16S rDNA). The primers (Helinest-S & R, Heli-S & R) used in our study were reported to amplify 26 species of *Helicobacter* genus [15].

#### First amplification

Amplification was carried out in a total volume of 50 µL containing 1 µg DNA, 25 µL DreamTaq™ Green PCR Master Mix (2 x) (Fermentas, CA, USA) and 50 pM of each primer (Helinest-S 5’-ATTAGTGGCGCACGGGTGAGTAA-3’ and Helinest-R 5’-TTTAGCATCCCGACTTAAGGC-3’). The reaction mixture was initially denatured for 2 minutes at 94 °C then amplified for 35 cycles as follows: denaturation for 30 seconds at 94 °C, primer annealing for 30 seconds at 55 °C and extension for 1.5 minutes at 72°C. A final extension step was done for 5 minutes at 72 °C (Thermocycler Robocycler Gradiant 96-STRATAGEN^®^, LA, USA).

#### Second amplification

Five microliter of amplicon from the first amplification step was used with primers Heli-S (5’-GAACCTTACCTAGGCTTGACATTG-3’) and Heli-R (5’**-**GGTGAGTACAAGACCCGGGAA-3’) and amplification was repeated as in the first amplification. PCR amplicons were visualised on a 2% agarose gel stained with ethidium bromide. The expected product size of the amplicon was 480 bp.

### DNA sequencing

PCR products were sequenced as described previously [15]. Sequence homology searches of the PCR products were performed with the Basic Local Alignment Search Tool (BLAST; National Center for Biotechnology Information).

### Statistical analysis

Data were analyzed using Statistical Package for the Social Sciences, Version 10 (SPSS Inc. Chicago, IL, USA). Different characteristics of study participants were described using mean ± SD, median (range) or number (percentage) as appropriate. Continuous variables were compared using the Student’s unpaired t-test or Mann-Whitney test. The association between categorical parameters was performed using the Chi-square test or Fisher’s exact test. P-values < 0.05 were considered statistically significant.

## Results

Table 1 summarizes the main demographic and laboratory characteristics of the 52 CHC patients. The mean age of patients was 42.5 ± 7.4 and only 5 were positive for anti-Schistosomal antibodies.

**Table 1:** Characteristics of the Chronic Hepatitis C Patients

Variables	N = 52
Age (year)^*^	42.5 ± 7.4
Gender^¶^	
Males	39 (75)
Females	13 (25)
ALT (IU/L)^*^	56.8 ± 32.5
AST (IU/L)^*^	50.7 ± 24.7
Albumin (g/dL)^*^	4.3 ± 0.51
Direct Bilirubin (mg/dL)^*^	0.27 ± 0.14
Total Bilirubin (mg/dL)^*^	0.72 ± 0.26
AFP (ng/ml)^#^	2.5 (0.1-33.7)
Hepatitis C Viral load (IU/mL)^#^	165 000 (4 320 - 3 520 000)
Anti-schistosomal Ab + ve^¶^	5 (9.6)
Fibrosis score	
Low	37 (71%)
High	15 (29%)

^*^mean ± SD; ^#^ median (range);^ ¶^ number (%).

Anti-*H. pylori* IgG was detected in the plasma of 31/52 (59.6%) patients. There were no significant differences in the demographic and laboratory characteristics among anti-*H. pylori* IgG-positive and anti-*H. pylori* IgG-negative patients (Table 2).

**Table 2:** Comparison Between Anti-*H. pylori* IgG Positive and Negative Patients Regarding Demographic and Laboratory Data

	Anti-*H.pylori* IgG + ve (n = 31)	Anti-*H.pylori* IgG - ve (n = 21)	P-value
Age (year)^a^	42.3 ± 6.9	42.9 ± 8.2	0.78
Gender^b^			
Male	24(77.4)	15 (71.4)	
Female	7(22.6)	6 (28.6)	0.75
ALT (IU/L)^a^	56.4 ± 27.5	57.4 ± 39.4	0.91
AST (IU/L)^a^	49.3 ± 20.7	52.8 ± 30.2	0.62
Albumin (g/dL)^a^	4.3 ± 0.5	4.4 ± 0.5	0.48
Direct bilirubin (mg/dL)^a^	0.25 ± 0.13	0.31 ± 0.15	0.13
Total bilirubin (mg/dL)^a^	0.72 ± 0.30	0.74 ± 0.2	0.79
AFP (ng/ml)^c^	2.9 (0.5 - 32.9)	2 (0.1 - 33.7)	0.48
HCV Viral load (IU/mL)^c^	95 700 (4 350 – 3 520 000)	225 000 (12 800 – 2 688 483)	0.18
Anti-schistosomal Ab + ve^b^	3 (9.7)	2 (9.5)	0.64
Fibrosis Scoring^b^			
Low stage	21 (67.7)	16 (76.2)	
High stage	10 (32.3)	5 (23.8)	0.51

^a^mean ± SD; ^b^number (%); ^c^median (range).

**Figure 1:**
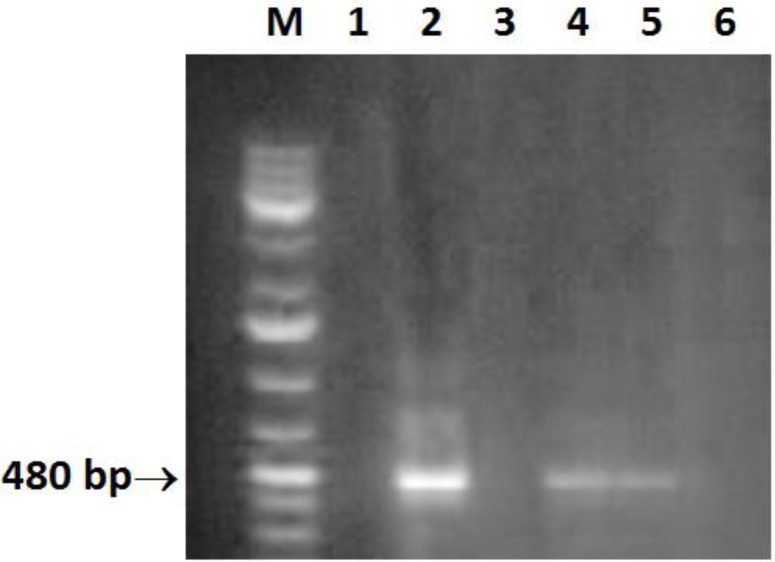
Amplification of a 480-bp 16S rRNA DNA fragment of Helicobacter. Lane M: molecular size marker (100 - 1000 bp); lane 2, 4, 5: positive samples; lane 6: negative control (double-distilled water).

*Helicobacter* DNA was present in 6/52 (11.5%) liver biopsy samples, collected from CHC patients, using *Helicobacter* genus specific 16 S rRNA gene primers (Fig. 1). The PCR products were sequenced and *H. pylori* like organisms were identified. All cases positive for *H. pylori* DNA in the tissue samples were positive for plasma anti-*H. pylori* IgG and negative for anti-schistosomal antibodies.

*Helicobacter* DNA positive cases were more frequent in CHC patients with high stage liver fibrosis 5/15(33.3%) than in low stage liver fibrosis 1/37 (2.7%). Thus, there was statistically significant (P = 0.0057) association between presence of *H. Pylori* DNA in the liver and stage of fibrosis. However, there was no association between the presence of *H. Pylori* DNA in the liver of CHC patients and age, gender, liver function tests, AFP levels or HCV viral load (Table 3).

**Table 3:** Comparison Between *H. pylori* DNA Positive and Negative Patients Regarding Demographic and Laboratory Data

	*H.Pylori* DNA + ve (n = 6)	*H.Pylori* DNA – ve (n = 46)	P-value
Age (year)^a^	45.6 ± 5.8	42.1 ± 7.5	0.28
Gender^b^			
Male	5 (83.3)	34 (74)	
Female	1 (16.7)	12 (26)	1.0
ALT (IU/L)^a^	64.3 ± 42.3	55.9 ± 31.5	0.56
AST (IU/L)^a^	57 ± 24.7	49.9 ± 24.9	0.51
Albumin (g/dL)^a^	4.6 ± 0.4	4.3 ± 0.5	0.17
Direct bilirubin (mg/dL)^a^	0.25 ± 0.14	0.28 ± 0.15	0.65
Total bilirubin (mg/dL)^a^	0.9 ± 0.47	0.7 ± 0.22	0.078
AFP (ng/mL)^c^	3.5 (0.5 - 32.9)	2.2 (0.1 - 33.7)	0.75
HCV Viral load (IU/mL)^c^	337 000 (51 200 - 3 520 000)	165 000 (4 320 – 2 688 483)	0.40
Fibrosis Scoring^b^			
Low stage	1 (16.7)	36 (78.3)	
High stage	5 (83.3)	10 (21.7)	0.0057

^a^mean ± SD; ^b^number (%); ^c^median (range).

## Discussion

Since the discovery of presence of *Helicobacter* species DNA in liver material from patients with liver disease, several studies were conducted to determine the role of these bacteria in the evolution of hepatic lesions to cirrhosis and HCC. Determinants of this evolution are not yet fully understood, including those occurring in HCV positive patients [10].

In the present study, *H. Pylori* DNA was detected in liver biopsies of 11.5% Egyptian patients with CHC which agrees with previous studies [10, 11, 16]. *H. pylori* DNA positive cases tended to be higher in CHC patients with high stage liver fibrosis (33.3%) than in low stage liver fibrosis (2.7%) (P = 0.0057). Our finding seems similar to the study of Castera et al in which they demonstrated higher prevalence of *Helicobacter* DNA in the liver samples from patients with hepatitis C cirrhosis than in those from HCV-infected patients without cirrhosis or from controls [16]. It has been suggested that *H. pylori* infection may affect the clearance of a concomitant viral infection through down regulation of the virus-specific T-lymphocyte helper-1 cytokine and T-lymphocyte suppressor responses [17]. Moreover, *H. pylori* DNA has been detected in the liver tissue of patients with cirrhosis and more frequently in HCC [10, 18]. The presence of *H. pylori* DNA at a higher frequency in the liver of patients with high stage fibrosis than low stage fibrosis may suggest the participation of this bacterium in the progress of the chronic hepatitis to HCC.

The fact that the DNA sequence, obtained from positive PCR of *Helicobacter* spp. specific 16 S rRNA gene, was analogous to *H. pylori* encourages the speculation that the presence of *Helicobacter* DNA in human liver tissue might reflect the transport of *H. pylori* of gastric origin or its DNA to the liver [15]. Verhoef et al reported that gastric colonization with a specific subset of *Helicobacter* strains is associated with the induction of HCC, either directly via colonization of the liver or indirectly via secretion of specific toxins by *Helicobacter* residing in the stomach [19]. Recently, oral administration of *H. pylori* to C57BL/6 mice, for two years, resulted in not only gastric lesions but also liver lesions, including inflammation, cirrhosis and hepatocyte hyperplasia with atypia. Thus, *H. pylori* inoculated orally could reach the liver and cause inflammation as an independent etiological factor [20].

In the present study, *H. pylori* DNA was found only in liver tissue from anti-*H. pylori* IgG positive patients that reflects stomach inoculation with *H. pylori* in these patients. This observation comes in agreement with several previous studies in which all *helicobacter* DNA positive liver samples were seropositive for *H. pylori* IgG [11, 16, 21]. The bacterium may pass from the stomach to the liver through the duodenum and biliary tract, or may arrive in the liver from the circulation through the hepatic portal vein [22]. Huang et al found *H. pylori* DNA in the peripheral blood of patients with duodenal ulcers infected with *H. pylori*, which suggested that *H. pylori* may spread to the liver by the blood-borne route [23].

This study also revealed the absence of any significant difference in liver function tests (ALT, AST, albumin, and bilirubin), AFP levels or HCV viral load among *H. pylori* DNA positive and *H. pylori* DNA negative patients. The lack of difference in liver function tests among these groups of patients indicates that the *Helicobacter* positivity may not primarily relate to severe liver damage. Our findings showed similarity with previous studies which, reported absence of association of any of these factors with respect to presence of *H. pylori* DNA in the liver of CHC and other chronic liver diseases patients [16, 24].

In conclusion, *H. pylori* DNA could be detected in liver of some CHC Egyptian patients and its presence is associated with more advanced liver fibrosis. Further studies are needed to investigate the role of *H. pylori* in the progression of chronic liver diseases to cirrhosis and its role in the development of HCC.
